# Evaluation of a Web-Based Medication Reconciliation Application Within a Primary Care Setting: Cluster-Randomized Controlled Trial

**DOI:** 10.2196/33488

**Published:** 2022-03-08

**Authors:** Michael R Gionfriddo, Yirui Hu, Bhumika Maddineni, Melissa Kern, Vanessa Hayduk, William R Kaledas, Nevan Elder, Jeffrey Border, Katie Frusciante, Maria Kobylinski, Eric A Wright

**Affiliations:** 1 Division of Pharmaceutical, Administrative and Social Sciences School of Pharmacy Duquesne University Pittsburgh, PA United States; 2 Center for Pharmacy Innovation and Outcomes Geisinger Health Danville, PA United States; 3 Department of Population Health Sciences Geisinger Health Danville, PA United States; 4 The Steele Institute for Health Innovation Geisinger Health Danville, PA United States; 5 Geisinger Medical Center Geisinger Health Danville, PA United States

**Keywords:** medication, reconciliation, electronic health record, information technology, medication safety, primary care, EHR, safety, app, randomized controlled trial, drug, interoperability, information source, mixed method, effectiveness, satisfaction

## Abstract

**Background:**

Despite routine review of medication lists during patient encounters, patients’ medication lists are often incomplete and not reflective of actual medication use. Contributing to this situation is the challenge of reconciling medication information from existing health records, along with external locations (eg, pharmacies, other provider/hospital records, and care facilities) and patient-reported use. Advances in the interoperability and digital collection of information provides a foundation for integration of these once disparate information sources.

**Objective:**

We aim to evaluate the effectiveness of and satisfaction with an electronic health record (EHR)-integrated web-based medication reconciliation application, MedTrue (MT).

**Methods:**

We conducted a cluster-randomized controlled trial of MT in 6 primary care clinics within an integrated health care delivery system. Our primary outcome was medication list accuracy, as determined by a pharmacist-collected best-possible medication history (BPMH). Patient and staff perspectives were evaluated through surveys and semistructured interviews.

**Results:**

Overall, 224 patients were recruited and underwent a BPMH with the pharmacist (n=118 [52.7%] usual care [UC], n=106 [47.3%] MT). For our primary outcome of medication list accuracy, 8 (7.5%) patients in the MT arm and 9 (7.6%) in the UC arm had 0 discrepancies (odds ratio=1.01, 95% CI 0.38-2.72, *P*=.98). The most common discrepancy identified was patients reporting no longer taking a medication (UC mean 2.48 vs MT mean 2.58, *P*=.21). Patients found MT easy to use and on average would highly recommend MT (average net promoter score=8/10). Staff found MT beneficial but difficult to implement.

**Conclusions:**

The use of a web-based application integrated into the EHR which combines EHR, patient-reported data, and pharmacy-dispensed data did not improve medication list accuracy among a population of primary care patients compared to UC but was well received by patients. Future studies should address the limitations of the current application and assess whether improved implementation strategies would impact the effectiveness of the application.

## Introduction

### Background

Nearly 50% of Americans are on at least 1 prescription medication [1]. Among Medicare patients, these prescriptions are prescribed on average by 7 different physicians [2]. This does not include nonprescription medications, such as over-the-counter medications, herbal medications, vitamins, and other supplements. The prescribing of medications from multiple providers and the prevalence of nonprescription medication use contribute to inaccurate medication lists. Inaccurate medication lists can lead to adverse consequences, including hospitalization and death [3]. This well-recognized problem has been the subject of initiatives from a variety of organizations [4-7] and is designated a National Patient Safety Goal by the Joint Commission [6].

Medication reconciliation has been recognized as problematic across a variety of health care settings, including primary care. Studies in primary care have found that the rate of discrepancies is high [8-10]. For example, we conducted a study within our primary care environment and identified that when asked, 369 (89.1%) of 414 patients requested a change to their medication list in the electronic health record (EHR) and, on average, patients noted 2.4 discrepancies on their medication lists [8]. At the time of this finding, there were few published tools to facilitate medication reconciliation in primary care and those that did had limitations. For example, a tool developed by Schnipper et al [11] was designed to be used after hospital discharge and compared the discharge medication list to the preadmission list held by the ambulatory care site. This tool was limited in that it was not applicable to all primary care patients and did not facilitate the collection of patient-reported medication use. Conversely, a tool by Lesselroth et al [12] collected medication information from patients using a kiosk; however, this technology was limited to Veterans Affairs facilities and may have missed medications not prescribed outside of those facilities.

To address the limitations of existing tools and improve medication reconciliation within our primary care environment, we developed an EHR-integrated web-based reconciliation application, MedTrue (MT), to facilitate the process of reconciling EHR medication lists with other sources, including patient-reported medications within a primary care environment. We designed this tool to have both staff- and patient-facing interfaces.

### Objectives

The goal of this paper is to present the results of an evaluation study aimed at assessing the impact of the application MT on medication list accuracy and patient and staff satisfaction. Using mixed methods, we aimed to evaluate the effectiveness of and satisfaction with MT.

## Methods

### Study Design

We conducted a mixed methods evaluation of MT, including a pragmatic cluster-randomized controlled trial (cRCT), along with patient and provider surveys and semistructured interviews. This study was approved by the Geisinger Institutional Review Board (2018-0174). Patient use of MT was considered not research as this technology was planned to become part of usual care (UC); however, the evaluative components were considered research.

### Setting

The trial was conducted at 6 primary care sites, with an even distribution of rural and urban clinics, within a large integrated health care delivery system. Within our system, there are over 40 primary care sites eligible to participate. The specific sites included were chosen collaboratively with input from primary care and clinic site leadership. Leadership was consulted to ensure alignment with ongoing initiatives and recommended sites based on their capacity to participate in the study (eg, adequate staffing). The included clinics had an average staff size (nurses/physicians/physician assistants) of 10. In the sites, nurses or medical assistants would bring the patient to the room, conduct a medication history and reconciliation, and perform a variety of other tasks to prepare the patient for a visit with their clinician. The clinician would then verify and approve any changes to the patient’s medication list. All sites used the same EHR, EpicCare® version 2018 (Madison, WI, USA).

### Population

Patients were eligible to participate if they were 18 years of age or older, able to speak English, and seen at a participating site by a member of the primary care team.

### Intervention

MT is a real-time web-based application that integrates information from the EHR, patients, and pharmacy-dispensed data into a common database that is viewable to patients through an online portal and in-clinic tablets and to clinicians through an EHR interface. The application was developed using an iterative design process. First, meetings were held with key stakeholders (eg, informatics, clinic staff, and pharmacy) to determine the necessary functionalities of the tool. Based on these initial requirements, we created a minimally viable prototype. We then iteratively improved upon this prototype by implementing it in 2 sites not involved in the later evaluation. These sites would use the tool and provide feedback to the study team. Additionally, study team members would observe the use of the tool in those sites to gather additional insight into how the tool was performing and what adjustments needed to be made. From the feedback received, we created a list of potential improvements, which was prioritized based on necessity and feasibility. The tool tested in this study is the result of several rounds of improvements.

**Figure 1:**
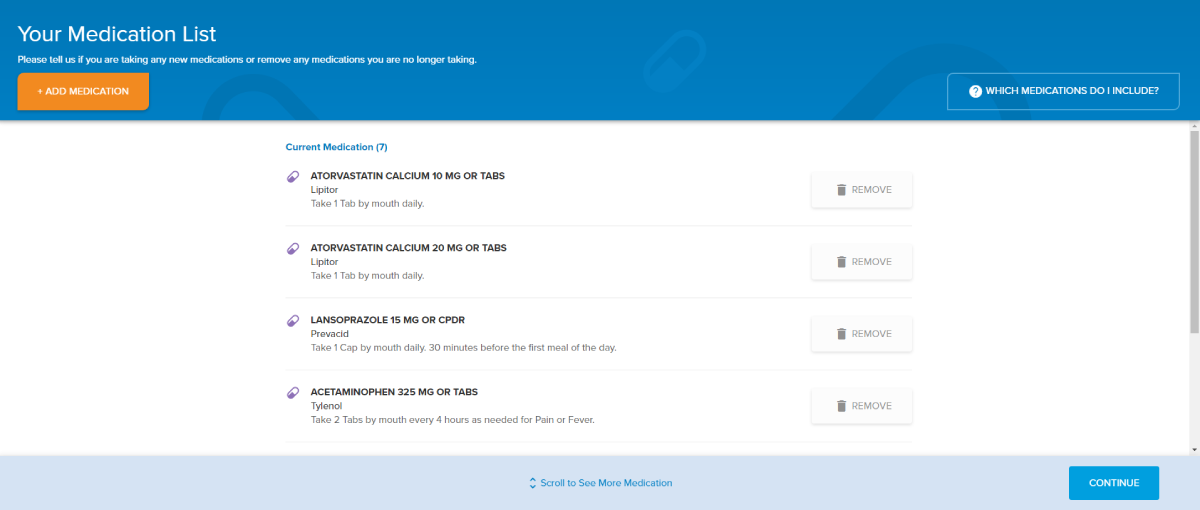
Patient interface.

The patient interface presents the patient with their active medication list that is located in the EHR, as well as pharmacy-dispensed data available as an additional function within the EHR (Figure 1). The patient is asked to review their list of medications, remove medications they are not currently taking, and enter additional medications missing from the list, such as medications purchased over the counter or medications they take that belong to a friend or family member. Additionally, patients are asked about their adherence to their medications over the past month using a Likert-type scale (ranging from 0% for “never” to 100% for “always”). This type of scale is similar to other validated scales for adherence [13,14]. Patients with an upcoming visit at 1 of the intervention sites had access to MT through our online patient portal up to 2 weeks prior to their visit. If the patient did not complete MT through the patient portal prior to their visit, they were provided with a tablet computer (iPad^®^) by patient access representatives upon check-in and were asked to complete MT while waiting for their appointment. Patients were not given any training in the use of the tool, since during prototyping, we found that most patients were able to navigate the tool without assistance. Approximately a third of the patients accessed MT using the online portal, while the remainder used iPads.

**Figure 2:**
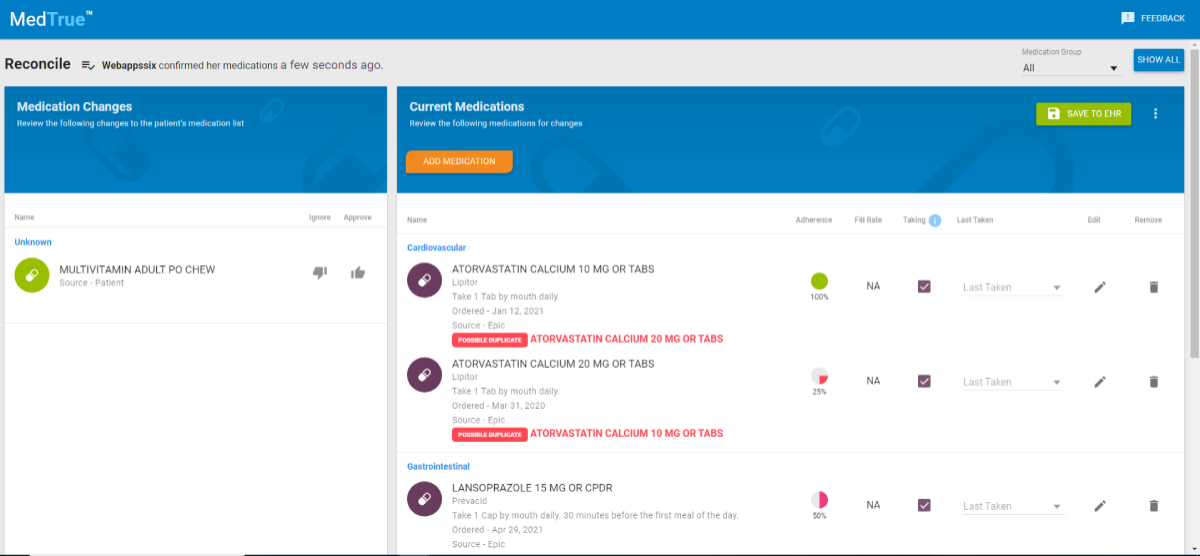
Staff interface.

MT was available to staff through a direct link (access tab) within the EHR as a web-based application. During the intake process and normal workflow of rooming a patient, staff (ie, nurses and medical assistants) accessed MT when assessing medication use. The interface was presented in an embedded browser within the EHR. For the staff interface, MT displayed all the medications the patient was presented with as well as those the patient entered (Figure 2). Discrepancies (ie, patient-suggested removals and additions or pharmacy-dispensed differences) were displayed alongside the EHR-listed medications that were not flagged as discrepant. For each medication adherence, calculated as the proportion of days covered from pharmacy-dispensed data [15], patient-reported medication adherence, captured from the patient-completed Likert-type item, was also displayed. Staff were instructed to conduct their medication history normally and use MT to confirm or change medications. All changes made within MT were “pushed” to the EHR upon clicking the “Save to EHR” button in MT. Staff at the intervention sites had access to paper and audiovisual training materials but also attended an in-person meeting where MT was introduced and reviewed by members of the study team. Study team members were available for consultation and guidance throughout the study.

### Usual Care

UC providers accessed the EHR-based medication profile that included the standard EHR list of active and discontinued/cancelled medications, along with a separate tab of medications collected through a feed of pharmacy-dispensed data. Patient-reported discrepancies (additions, changes, etc) were also available if the patient completed a previsit questionnaire through the online patient portal (approximately a third of patients have portal accessibility). Standard medication list patient information collected was similar to the MT intervention but did not include self-reported adherence. Hence, UC clinics also had a degree of patient and pharmacy-dispensed information available to clinicians, along with EHR lists, although this information was not available for all patients and information in the EHR was not available within 1 tab. The EHR system used in both UC and MT clinics was EpicCare® version 2018.

### Data Collection

#### Best-Possible Medication History

Trained pharmacists approached a convenience sample of patients exposed to MT as well as a convenience sample of patients in the UC arm to collect a best-possible medication history (BPMH); feasibility considerations informed our decision to use convenience sampling. All pharmacists were licensed and had experience conducting medication histories. Prior to enrolling patients, pharmacists underwent brief training by study staff reviewing components of a BPMH and a clinical interview guide complete with prompts (Multimedia Appendix 1). This guide was based on the literature related to conducting a BPMH [7,16,17]. Pharmacists identified patients via a custom web-based dashboard listing patients with a documented launch of MT by a clinician (MT arm only) or arrival to an eligible visit for UC. Patients completing an encounter in UC were assumed to have EHR-based medication reconciliation due to routine workflow completion of medication reconciliation in all primary care encounters. Pharmacists were blinded to clinic site use of MT, as dashboards only supplied names, not information about MT use. Only study personnel and clinic staff were aware of study group assignment. Pharmacists approached eligible patients after their visit and asked whether they would be interested in participating in a study. If a patient was agreeable, the pharmacist brought them to a private room for consent and to conduct the BPMH.

Pharmacists documented discrepancies for each medication on the postvisit EHR active medication list and also for any medication not present on the medication list. Each medication was coded with only 1 type of medication discrepancy, which fell into 1 of 4 domains. The first was patient acknowledgement of taking the medication differently than prescribed (eg, allergy medication is prescribed daily, but the patient takes it seasonally). Second was removal of a medication on the medication list. The third domain was a modification to the formulation, strength, dose, frequency, route of medication, or timing of administration. The final discrepancy was adding a medication to a profile that was not on the EHR medication list. Since taking a medication differently than prescribed would not automatically infer an inaccurate medication or description of the medication on the medication profile (eg, the patient reported missing a weekend dose of a medication due to forgetfulness), we did not include this in our composite discrepancy endpoint (see later) but did report on this individually. In addition, after each BPMH, pharmacists provided feedback on their confidence that the list they gathered from the patient was accurate using a 3-point scale (not confident, somewhat confident, and confident).

Following the final BPMH collection, study investigators reviewed the reconciled lists of medications in a blinded manner to ensure consistent coding of discrepancies. Inconsistencies were flagged and recoded for final analysis.

Consent, the BPMH, and pharmacist questions were documented using REDCap (Vanderbilt University, Nashville, TN, USA) [18,19]. The interviews took up to an hour, and patients were compensated US $10 for their time in the form of a gift card.

#### Surveys

All patients exposed to MT were given an opportunity to complete a survey on the usability of and satisfaction with using MT. The survey was presented to the patient following MT completion. Responses were collected within MT.

Staff participating in the intervention arm of the study were also given a survey on the usability of and satisfaction with using MT near the end of the study. Staff were contacted by email and invited to participate in the survey, with up to 2 reminder emails sent at weekly intervals. Surveys were collected using Microsoft Forms (Microsoft Corporation, Redmond, WA, USA).

#### Interviews

The interviews were guided by a qualitative descriptive approach [20]. Patients in the intervention arm were invited to participate in interviews about their experience using MT through convenience sampling. After a visit where MT was used, patients were approached by a research assistant, who invited them to participate in a brief interview. Consenting patients were interviewed using a semistructured interview guide (Multimedia Appendix 1). The research assistant underwent brief training in qualitative interviewing from 1 of the investigators with experience in qualitative interviewing. The interviews lasted no more than 30 minutes, and patients were compensated US $10 for their time in the form of a gift card.

Rooming staff who used MT during the study period were also given an opportunity to voluntarily participate in semistructured interviews to discuss their experience using MT. The interviews were conducted by an investigator with experience in qualitative interviewing using a semistructured interview guide (Multimedia Appendix 1). The interviews lasted no more than 30 minutes, and staff were not offered compensation.

### Primary Outcome

The primary outcome of the study was the accuracy of the medication list. We defined accuracy by counting discrepancies between the medication list gathered through MT or UC and the list gathered by the pharmacist conducting the BPMH. A perfectly accurate medication list would be a list with 0 identified discrepancies.

### Secondary Outcomes

We secondarily measured accuracy using a composite numerical assessment of accuracy, where each medication in a list could contribute either 0 or 1 discrepancy, and all discrepancies were totaled per patient. This endpoint included all of the following medication discrepancies: not taking the listed medication, needing the medication to be modified (eg, change in dose or frequency), or having the medication added to their list. Although taking a medication differently was included in our list of discrepancies, this was not included in the composite accuracy list, since this discrepancy would in of itself not necessitate a change in the actual prescription as written, and if the list needed to be modified by the BPMH, 1 of the other listed discrepancies would have been selected over this discrepancy, as per BPMH instructions. We reported individual types of discrepancies identified per group (ie, adding, modifying, removing, or taking a medication differently) on a medication level. To determine the rate of medication discrepancies per patient, we calculated the percentage of the patients’ medications that had discrepancies. Finally, we conducted a sensitivity analysis based on the pharmacists’ confidence in their BPMH, including only those patients where the pharmacist conducting the BPMH was confident in having an accurate medication list. Patient and staff satisfaction with MT, as measured by surveys and qualitative interviews, was also reported.

### Sample Size

Assuming a 1% intracluster correlation coefficient and a 19% difference in accuracy rates, our target sample size was 300 patients (n=150 [50%] intervention, n=150 [50%] control; each of the 6 sites was expected to enroll 50 [16.7%] participants) to achieve 80% power. During data collection, we conducted an interim look to verify our sample size assumptions and found that our initial assumptions regarding the proportion of completely accurate medication lists (no discrepancies), which were based upon previous studies [21-23], were overly optimistic (~50% in previous studies vs 4% in our interim analysis). As a result, we were underpowered to detect our primary outcome; however, we were powered to detect a secondary composite outcome based on a count of discrepancies. Later, due to feasibility, namely difficulties in recruitment, the trial was stopped.

### Randomization

In total, 6 sites were chosen in collaboration with primary care leadership and randomized to the intervention or the control arm using a random number generator in Microsoft Excel (Microsoft Corporation). Prior to recruitment, due to unforeseen challenges, 1 UC site was substituted for a different clinic with similar baseline characteristics.

### Blinding

Neither patients nor clinicians were blinded due to the nature of the intervention; however, there were steps taken to blind the pharmacists conducting the BPMH (as described in the Data Collection section). During the analysis, the statistician was blinded as to which arm was intervention and which was control.

### Analysis

#### Qualitative Analysis

Interviews were transcribed verbatim and analyzed thematically [24]. Patient and staff interviews were separately analyzed. The transcripts were independently read by 2 study team members, who collaboratively developed 2 codebooks (1 for patients and 1 for staff). The transcripts were independently coded by 2 study team members using the appropriate codebook. Discrepancies in coding were discussed and resolved by consensus. The codes were then grouped into themes, which were refined based on discussion.

#### Statistical Analysis

To understand the representativeness of our sample, we compared the demographics of patients seen at the clinics during the intervention period to those who received a BPMH. We described continuous variables using means and SDs as well as medians and IQRs. Categorical variables were described using frequencies and percentages. Wilcoxon rank-sum tests were used to compare the differences between 2 groups for skewed continuous outcomes; chi-square tests were used to evaluate the independence of categorical outcomes between MT and UC, where odds ratios (ORs) were presented. A mixed effects model with Poisson regression was performed to model the impact of MT on count data (number of medication discrepancies) after adjusting for age, sex, and Charlson comorbidity index, with the total number of medications as the offset variable. Incidence rate ratios (IRRs) and 95% CIs were estimated from Poisson regression models. Sensitivity analyses were performed with or without considering cluster-level covariates for cRCTs. *P*<.05 was considered statistically significant. Statistical analyses were performed using RStudio version 1.2.1335 (RStudio, PBC, Boston, MA, USA) [25].

Additionally, we conducted sensitivity analyses including only those patients for whom the pharmacist rated that they were confident in the history they gathered from the patients; histories where the pharmacist was either somewhat confident or not confident were excluded.

## Results

### Patient Characteristics

MT was implemented in 3 clinics from July 10, 2018, through August 1, 2019. Over this time frame, MT was accessed 10,835 times by 7342 distinct patients. The majority of access (6501/10,835, 60%) was by tablet computer. Of the 7342 patients using MT, 4005 (54.55%) removed at least 1 medication (mean 3.6 medications removed), 606 (8.25%) added at least 1 medication (mean 1.6 medications added), and 6061 (82.55%) completed the adherence questions. Staff accessed MT for 8443 distinct patients (staff could access MT even if patients did not), removed at least 1 medication for 3544 (41.98%) patients (mean 2.1 medications removed) and added at least 1 medication for 1643 (19.46%) patients (mean 3.3 medications added).

Clinics allocated to MT and UC had similar baseline characteristics, although patients at MT clinics were slightly younger in age (46 vs 48 years), had fewer African Americans (n=871 [3%] vs n=1255 [5%]), less depression (n=1441 [5%] vs n=1490 [6%]), and took more medications (7.75 vs 6.53 medications per patient); see Table 1.

Overall, 224 patients were recruited and underwent a BPMH with the pharmacist (n=106 [47.3%] MT, n=118 [52.7%] UC). The samples of patients who underwent a BPMH were older; were more likely to be female; had depression, dementia, or mild cognitive impairment; were more likely to be married; and had more medications than the baseline clinic population (similar between groups).

**Table 1:** Demographics.

Characteristic		MT^a^ (clinic level), N=30,275	UC^b^ (clinic level), N=26,463	*P* value (MT vs UC population)	UC (patient level), N=118	*P* value (population vs sample)	MT patient level), N=106	*P* value (population vs sample)	*P* value (MT vs UC sample)
**Age (years), mean (SD)**		46 (23)	48 (21)	<.001	55 (18)	<.001	52 (18)	.01	.23
**Female, n (%)**		15,740 (59.48)	17,886 (59.08)	.35	76 (64.41)	.04	71 (66.98)	.10	.69
**Race, n (%)**				<.001		.02		.54	.79
	Caucasian	29,021 (95.86)	24,882 (94.03)		114 (96.6)		104 (95.3)		
	African American	871 (2.88)	1255 (4.74)		3 (2.5)		3 (2.8)		
	Other	383 (1.27)	326 (1.23)		1 (0.8)		2 (1.9)		
**Ethnicity: Hispanic, n (%)**		1243 (4)	1003 (4)	.06	4 (3)	.03	2 (2)	.03	.49
**Charlson comorbidity index, mean (SD)**		2.20 (2.79)	2.37 (2.84)	<.001	3.12 (2.92)	<.001	2.64 (2.72)	.01	.25
**Disorder, n (%)**									
	MCI or dementia	350 (1.16)	372 (1.41)	.01	5 (4.2)	.01	5 (4.7)	<.001	.86
	Depression	1441 (4.76)	1490 (5.63)	<.001	23 (19.49)	<.001	25 (23.58)	<.001	.46
**Relationship status (married), n (%)**		1682 (5.56)	1478 (5.59)	.88	63 (53.4)	<.001	47 (44.3)	<.001	.18
**Number of medications, mean (SD)**		7.75 (5.64)	6.53 (5.05)	<.001	12.38 (7.54)	<.001	11.15 (7.29)	<.001	.16

^a^MT: MedTrue.

^b^UC: usual care.

### Primary Outcome

For our primary outcome of medication list accuracy (defined as 0 discrepancies), we did not detect a statistically significant difference between MT and UC (OR=1.01, 95% CI 0.38-2.70, *P*=.98). Pharmacists completing the BPMH reported being confident in the medication history for 200 (89.2%) of 224 patients (91 [85.8%] MT, 109 [92.4%] UC). The primary outcome remained the same upon conducting a sensitivity analysis limited to those BPMHs in which the pharmacist was confident (OR=1.08, 95% CI 0.39-3.02, *P*=.88).

### Secondary Outcomes

The composite secondary outcome of total medication discrepancies, defined as the sum of additions, medications the patient was not taking, and medications that were modified summed per patient, was comparable between MT and UC arms (MT median=4, IQR 2-7 vs UC median=3, IQR 2-6; *P*=.15). The most common discrepancy was medication the patients’ reported not taking, with a median of 2 (IQR 1-3) identified per patient in the UC arm and 1 (IQR 1-3) per patient identified in the MT arm.

The composite outcome was consistent upon sensitivity analysis limited to those patients where a pharmacist was confident in their BPMH (MT median=3, IQR 2-5 vs UC median=4, IQR 2-7; *P*=.10).

A Poisson regression model indicated that the MT and UC arms were comparable regarding the rate of discrepancies (IRR=1.22, 95% CI 0.97-1.55, *P*=.09). The rate of discrepancies was significantly affected by age (IRR=1.01, 95% CI 1.00-1.01, *P*=.03), male sex (IRR=0.81, 95% CI 0.71-0.93, *P*=.003), and Charlson comorbidity index (IRR=0.95, 95% CI 0.92-0.98, *P*=.001). Sensitivity analyses with or without considering cluster-level covariates confirmed the robustness of findings of the Poisson regression model (data not shown).

In addition to the composite, we assessed accuracy as the proportion of medications that were discrepant. The proportion of discrepant medications was not significantly affected by MT compared to UC (MT median=40%, IQR 23%-54% vs UC median=34%, IQR 21%-59%; *P*=.87); this finding was similar on sensitivity analysis (MT median=40%, IQR 22%-55% vs UC median=33%, IQR 21%-56%; *P*=.66). Further accuracy data can be found in Tables 2-4.

**Table 2:** Number of patients, with discrepancy type.

Medication discrepancies		UC^a^ (N=118), frequency (%)	MT^b^ (N=106), frequency (%)	OR^c^ (95% CI)	*P* value^d^
**Primary outcome^e^**		109 (92.4)	98 (92.5)	1.01 (0.38-2.72)	.98
	Modification	72 (61.0)	55 (51.9)	0.69 (0.41-1.17)	.17
	Addition	51 (43.2)	42 (39.6)	0.86 (0.51-1.47)	.59
	Not taking medication	93 (78.8)	79 (74.5)	0.79 (0.42-1.46)	.45
**Taking medications differently**		24 (20.3)	22 (20.8)	1.03 (0.54-1.96)	.94

^a^UC: usual care.

^b^MT: MedTrue.

^c^OR: odds ratio.

^d^*P* value based on chi-square test.

^e^Primary outcome including not taking + modifying + adding medications.

**Table 3:** Total number of discrepancies per patient.

Medication discrepancies		UC^a^ (N=118), mean (SD)	MT^b^ (N=106), mean (SD)	UC (N=118), median (IQR)	MT (N=106), median (IQR)	*P* value^c^
**Primary outcome^d^**		4.84 (3.85)	4.58 (4.89)	4 (2-7)	3 (2-6)	.15
	Modification	1.51 (1.84)	1.29 (1.96)	1 (0-2)	1 (0-2)	.17
	Addition	0.85 (1.33)	0.72 (1.23)	0 (0-1)	0 (0-1)	.43
	Not taking medication	2.48 (2.60)	2.58 (4.10)	2 (1-3)	1 (0-3)	.21
**Taking medications differently**		0.33 (0.77)	0.28 (0.61)	0 (0-0)	0 (0-0)	.92

^a^UC: usual care.

^b^MT: MedTrue.

^c^*P* value based on Wilcoxon test.

^d^Primary outcome including not taking + modifying + adding medications.

**Table 4:** Percentage of medications on the list discrepant.

Medication discrepancies		MT^a^ list %, mean (SD)	UC^b^ list %, mean (SD)	MT list %, median (IQR)	UC list %, median (IQR)	*P* value^c^
**Primary outcome^d^**		40 (23)	39 (24)	40 (23-54)	34 (21-59)	.87
	Modification	11 (13)	10 (14)	8 (0-18)	6 (0-17)	.36
	Addition	8 (14)	8 (14)	0 (0-12)	0 (0-11)	.68
	Not taking medication	20 (17)	21 (21)	18 (6-29)	16 (1-33)	.82
**Taking medications differently**		3 (8)	4 (9)	0	0	.63

^a^MT: MedTrue.

^b^UC: usual care.

^c^*P* value based on Wilcoxon test.

^d^Primary outcome including not taking + modifying + adding medications.

### Usability and Satisfaction

Of the 7342 patients who accessed MT, 1450 (19.75% response rate) completed the survey. Patients were satisfied with MT, giving it an average rating of 8 out of 10 for their likelihood to recommend to a friend or family member (Table 5).

Additionally, 1276 (88%) of 1450 patients agreed or strongly agreed that it was easy to use, and 1233 (85%) agreed or strongly agreed that it helped them create a more accurate medication list (Table 6).

**Table 5:** Patient net promoter score (N=1450).

Patient survey question	Score (1-10)										Mean (SD)
	1, n (%)	2, n (%)	3, n (%)	4, n (%)	5, n (%)	6, n (%)	7, n (%)	8, n (%)	9, n (%)	10, n (%)	
*On a scale of 1 (low) to 10 (high), what is the likelihood you would recommend MT^a^ to a friend or family member?*	41 (2.8)	15 (1)	15 (1)	18 (1.2)	155 (10.7)	112 (7.7)	149 (10.3)	222 (15.3)	156 (10.8)	567 (39.1)	7.95 (2.29)

^a^MT: MedTrue.

**Table 6:** Patient survey data (N=1450).

Patient survey	Strongly agree, n (%)	Agree, n (%)	Neutral, n (%)	Disagree, n (%)	Strongly disagree, n (%)
*I would use MT^a^ in the future.*	643 (44.3)	568 (39.2)	169 (11.7)	24 (1.7)	46 (3.2)
*MT helped me create an accurate medication list.*	641 (44.2)	596 (41.1)	124 (8.6)	44 (3.03)	45 (3.1)
*MT is easy to use.*	724 (49.9)	559 (38.6)	103 (7.1)	17 (1.2)	47 (3.2)
*MT reminded me to include all my medications, including those my doctor prescribes and those I purchase over the counter.*	721 (49.7)	592 (40.8)	77 (5.3)	21 (1.4)	39 (2.7)

^a^MT: MedTrue.

These findings were echoed in the patients’ qualitative interviews and were consistent across sites. Of the 7342 patients who used MT, 14 (0.2%) participated in interviews (1 spouse was also present for 1 of the interviews). Overall, participants had a positive experience with MT. Most found it easy to use and were able to complete the application quickly. The ability to visually see their list of medications rather than be asked about them was noted as an important feature. Seeing the medication names helped patients recognize the medications because they are used to seeing them in print on their medication packaging. Some patients felt it was more efficient than the normal workflow, whereas others disagreed and felt it was creating unnecessary work for them. The patients also noted that having the indication for the medication would help with recognition and recall. Example quotes from patients illustrating these themes can be found in Table 7.

Of the 22 rooming staff members who accessed MT, 18 (82% response rate) completed the survey. Staff were unsatisfied with MT, giving it an average rating of 0 out of 10 (Table 8).

All staff members disagreed or strongly disagreed that they were satisfied with MT and noted that it did not fit well in their workflow (Table 9).

Interviews provided a more nuanced perspective from staff, with themes and example quotes in Table 10.

**Table 7:** Patient feedback on MTM^a^ (N=14).

MT characteristic	Example quote
Usability	*. . . It’s that simple. I mean, I think somebody from 9 to 90 can use it. I really, I really do, because it’s . . . there’s nothing confusing about it. It was, and most things are to me, but it truly wasn’t. It was quicker than I thought it would be. I thought we’d be there for a while answering questions after. But it wasn’t; it was simple.* [Patient T12]
Benefits	*I think it did help. Because normally, checking in, they would ask what I’m currently taking. This also showed what I was taking 4 months ago but I’m not now. That wouldn’t have even crossed my mind if it wasn’t right in front of me. I would have never thought to say, “Oh, I’m no longer taking the clobetasol. Oh, I’m not on prednisone anymore.” But because it was right in front of me, I thought, oh yeah, I can remove that. I can remove that. I found that to be helpful.* [Patient T13] *I thought it was easy, and I like that I can delete things off of there if I want. Because I’ve been askingfor 1 medication to be taken off and it hadn’t, so I was able to do that.* [Patient P7] *It’s just time-saving. I mean, you’re going to sit there and wait for the doctor anyway, so you might as well get it done. And then, it makes it quicker once you’re back in the room. You have control over what’s in your chart—which I feel like, sometimes, you don’t, and, you know, you go to another doctor, and they’re like, “Oh, I see you’re taking this.” And it’s like, I haven’t taken that in a million years, you know?* [Patient P7]
Necessity	*. . . It’s just an extra thing to do on our part, and I really wonder if it’s really necessary to get that information.* [Patient T9]
Suggestions	*On the meds, the listings of the meds, you’d had both the marketing name and you have the commonly known. The other thing, if you could do it simple, after that say, you know, blood thinner or cholesterol drug for the average person. That’s what they think about it. “Oh, that’s my blood pressure med. That’s my cholesterol med.” Because if not, they look at it, you know, hydrochlorothiazide. They’re like, “I don’t know what that is.”* [Patient T8]

^a^MT: MedTrue.

**Table 8:** Staff net promoter score (N=18).

Staff survey question	Score (1-10)										Mean (SD)
	1, n (%)	2, n (%)	3, n (%)	4, n (%)	5, n (%)	6, n (%)	7, n (%)	8, n (%)	9, n (%)	10, n (%)	
*On a scale of 1 (low) to 10 (high), what is the likelihood you would recommend MT^a^ to a friend or family member?*	16 (88)	1 (6)	—^b^	—	1 (6)	—	—	—	—	—	0.28 (0.96)

^a^MT: MedTrue.

^b^Not applicable.

**Table 9:** Staff survey data (N=18).

Staff survey	Strongly agree, n (%)	Agree, n (%)	Neutral, n (%)	Disagree, n (%)	Strongly disagree, n (%)
*I am satisfied with MT* ^a^ *.*	—^b^	—	—	4 (22)	14 (78)
*I want to use MT in the future.*	—	—	—	3 (17)	15 (83)
*I would want to use MT at every visit with every patient.*	—	—	—	3 (17)	15 (83)
*MT helped me conduct medication reconciliation with my patients.*	—	—	—	6 (33)	12 (67)
*MT is compatible with my clinic flow.*	—	—	—	3 (17)	15 (83)
*MT is easy to use.*	—	1 (6)	—	3 (17)	14 (78)

^a^MT: MedTrue.

^b^Not applicable.

**Table 10:** Staff feedback on MT^a^ (N=11).

MT characteristic	Example quote
Usability	*Yeah, like overall, if it wasn’t so slow and it didn’t refresh after every time you clicked something, there really would’ve been, like, no, like, huge issue with it; like, it was just the fact that, like, every time you clicked a box, it just spun—which, like, you know, really held you up.* [Nurse P4] *Oh, it was very usable. It was very easy. Like again minus the slow Wi-Fi once we got it up and running it was like very easy to get through and you know kind of clear, to the point, you can click on things very easily.* [Nurse P1]
Benefits	*I’ve been able to say, well, you know, you say you’re taking your Lasix, but it looks like you’ve only been getting it filled, like, 60% of the time. Is there a reason, you know, that you weren’t getting it filled? Because I’m concerned that maybe you aren’t taking it like you should. So, it opened up that dialogue . . .* [Nurse MT9] *I think the benefitsof MT are we get a more accurate picture of the medications patients are on, which in the long run helps us treat them better.* [Nurse MT9]
Functionality	*MT was not able to do that. Um, in [the medical record], they have, like, a little Post-It. It looks like a Post-It note next to the medication, and you just click on that, and you can enter in a comment. And I can say [the] patient has not started yet or will start or you know was too expensive.* [Nurse P1]
Patient Concerns	*They will remove something in the waiting room, and then they get frustrated that we’re still going over it with them in the room.* [Nurse MT7]

^a^MT: MedTrue.

## Discussion

### Principal Results

The use of MT, a web-based application facilitating medication reconciliation, did not impact the accuracy of medication lists. The application was well received by patients; however, implementation challenges limited usability and acceptance by staff. The failure to improve medication list accuracy reflects both implementation challenges as well as the challenge of reliance on a technology-only solution to obtaining accurate medication lists.

### Comparison With Prior Work

Our application is 1 of many technologies and applications being developed to obtain and maintain accurate medication lists [26-32]. Many applications are focused on hospitalized patients, and not all applications have been pragmatically evaluated or included a methodology to compare accuracy with the gold-standard BPMH. Lesselroth et al [12,33] have developed a medication reconciliation kiosk where patients can review and edit their medication lists. Similar to MT, this information is imported into the EHR and reviewed by clinicians [31]. In their evaluation, they found similar rates of discrepancies (29%-39% vs 34%-40%), and the overall number of patients with discrepancies were similar (91%-99% vs 92%-93%) [33,34]. When evaluating the implementation of their application, they also found that time is a factor in using medication reconciliation technology and that, to be taken up, the technology needs to be well integrated into current workflows [35]. This was also found to be a challenge with our application despite its intent to work within the staff workflow.

### Strengths

We implemented several design elements to enhance the validity of our evaluation. We randomized clinics to MT or UC to avoid potential contamination that might have occurred at the patient or clinician level. We controlled for possible confounding factors through an adjusted analysis, the results of which mirrored the unadjusted analysis, increasing our confidence in our results. Throughout the trial, we also attempted blinding, wherever possible, to minimize bias. Other strengths include the use of pharmacists to conduct a BPMH and our application itself, which incorporates patient, clinician, and pharmacy-dispensed data to facilitate medication reconciliation.

### Limitations

Despite these strengths, a few limitations should be noted. Due to the nature of the intervention, clinicians and, to an extent, patients could not be blinded to the intervention. Fidelity to the use of MT was not always optimal and was variably used among rooming staff. As indicated in the staff survey and interviews, MT was not favorably viewed by the staff and as such they might have failed to use MT consistently, which would reduce observed effectiveness. Technical challenges, such as loading delays and internet connectivity for tablets, also affected the usability of the application in our clinics. Additionally, although the application was implemented within the EHR, it was outside of the existing workflow and required several additional clicks. Finally, we are underpowered to confidently detect the differences we observed in this trial. This was, in part, due to optimistic initial estimates of the effect of MT, as well as recruitment challenges. Our initial estimates of the effect of MT may have been optimistic as by the time the application was implemented, certain features were also available in the EHR, thereby reducing the difference between MT and UC.

### Conclusion

Novel approaches to medication reconciliation are needed to ensure safe and effective medication use. The use of technology, such as MT, a web-based application integrating various data sources, is a promising solution to reduce the rate of discrepancies. In our study, MT did not affect the rate of discrepancies, which may have been the result of failure to use the tool consistently and with fidelity due to implementation challenges. Thus, technology solutions may need to be paired with additional implementation strategies, and particular attention should be paid to how these technologies and other innovations are integrated into clinical workflows to facilitate fidelity and maximize effectiveness.

## Appendix

Multimedia Appendix 1 Qualitative interview guides.

Multimedia Appendix 2 CONSORT-EHEALTH checklist (V 1.6.1).

## Abbreviations

BPMH: best-possible medication history
cRCT: cluster-randomized controlled trial
EHR: electronic health record
IRR: incidence rate ratio
MT: MedTrue
OR: odds ratio
UC: usual care
