# Supplementary material for: Evaluation of a Web-Based Medication Reconciliation Application Within a Primary Care Setting: Cluster-Randomized Controlled Trial
Source: JMIR Form Res. 2022 Mar 8;6(3):e33488. doi: 10.2196/33488 (PMC8941436; doi:10.2196/33488)
Supplement: Multimedia Appendix 1 [file formative_v6i3e33488_app1.docx]

**APPENDIX**

**Pharmacist Clinical Interview Guide**

**I. Opening**

Pharmacist greets the patient.

Example opening

“Good afternoon. My name is [insert name here]. You may call me [insert preferred name], what would you like me to refer to you by? I am a pharmacist and I am working with Geisinger to better understand the medications that you are taking. Are you interested with spending about an hour with me, talking about your medications?”

**II. Consent**

Pharmacist reviews the written consent document and HIPAA Authorization with the patient.

[Please see written consent document for further description]

**III. Clinical Interview**

Upon consenting the patient, the pharmacist will begin the clinical interview.

1. Confirm the patient’s identity
   1. Name
   2. Date of birth
2. Access patient’s medication history
3. Review patient’s medication list with the patient
   1. Please tell me how you are taking your medication?
      1. Dosage
      2. Dose
      3. Frequency
      4. Dosage form
      5. Route
      6. Last dose taken?
   2. If patient not taking a medication: What is the reason for deviating from the dosage, frequency, dosage form or for not taking the medicine at all?
   3. Are you using any other prescription medications that are not mentioned on this list?
      1. Any injectable medications (e.g. insulin)
   4. Are you using non-prescription medications (e.g. medications sold over the counter) (If so, go back to prompt a above)
      1. Medications (e.g. aspirin, medications for heartburn, pain, etc.)
      2. Vitamins (e.g. multi-vitamin)
      3. Supplements (e.g. iron or calcium or glucosamine)
      4. Creams, lotions, or ointments?
      5. Cough drops or cough syrups?
   5. Are you using homeopathic drugs or herbal medicines (e.g. St. John’s wort)?(If so, go back to prompt a above)
   6. Are you using medications that belong to family members or friends?
      1. If yes, why?
   7. Are you using any ‘‘as needed’’ medications (i.e. medications that you only take once and awhile)?
   8. Does anyone ever help you take your medications?
      1. Who?
      2. Which ones?
      3. How often?
   9. Do you use a pillbox or similar device?
      1. Does anyone help you fill it?
         1. Who assists you?
         2. Under what circumstances?
   10. Are you experiencing problems taking your medication?
   11. In case of inhalation therapy: What kind of inhalation system are you using (e.g. inhalers, nasal sprays)?
       1. Are you experiencing any problems using this system?
   12. In case of eye or ear drops: Are you experiencing any difficulties using the eye drops?
   13. Do you ever forget to take your medication? If yes, which medication? Why? What do you do?
   14. Do you have any drug allergies? If yes, specify the drugs/drug classes and symptoms of the allergy
   15. Do you have any drug intolerances? If yes, specify which drugs/drug classes and symptoms of the intolerance
4. Is there anything else you would like to tell us about your medication or is there anything we missed?

**Closing**

Thank the patient for their participation, give an opportunity for any final questions, and remind the patient that they should contact their doctor with any concerns about their medication, and if they have concerns about the study they can contact the study team or the IRB.

**Notes**

If questions come up during the clinical interview regarding the patient’s medication list, the pharmacist may call the patients pharmacy for confirmation or clarification if agreed to by the patient.

**Semi-Structured Patient Follow-up Interview Guide**

**Opening**

Welcome patient, thank them for volunteering, introduce self and staff (if present), confirm with patient that the session may be recorded, and review oral consent.

The purpose of the project overall is to better understand your medications. What we are asking of you today is to share your thoughts and experiences using the MedTrue™ application and teach us about that experience. This project is a collaboration between Merck and Geisinger.

**Oral Consent**

You have recently attended a visit with a Geisinger clinician. As part of that visit you used a tool to help use better understand your medication use, a tool called MedTrue™. After completing the tool you answered a question indicating that you would be willing to discuss you experience using MedTrue™. You can choose not to answer any questions you do not feel comfortable answering.

The interview will last about a half hour and will be audio recorded. At all times, you will know where the recorder is and if it is turned on. At any time, you can ask to stop and turn off the recorder. Once we are done with the interview the recording will be moved to a secure and password protected server only accessible by authorized study team members. This recording will then be deleted from the recorder. We will then transcribe the recording removing any statements or data which could identify you. Upon checking the transcription for accuracy, we will delete the recording stored on our servers.

There are no known risks to you from taking part in this research study.

Although you may not directly benefit from this research, there is a potential benefit to people in the future as a result of information gathered in the research study.

You will receive a $10 gift card for participating in this research.

Please understand your participation is voluntary and you have the right to withdraw your consent or discontinue participation at any time without penalty. Specifically, your current or future care at Geisinger will not be jeopardized if you choose not to participate.

If you have any questions about this research study you can contact Dr. Eric Wright at XXX-XXX- XXXX or Dr. Michael Gionfriddo at XXX-XXX-XXXX.

If you would like to obtain more information, offer input or discuss problems or concerns about your rights as a research participant, please call Geisinger Institutional Review Board (IRB) at XXX-XXX-XXXX or XXX-XXX-XXXX, referencing IRB # 2018-0174.

**Questions**

General

1. Tell me about your experience using MedTrue™?
   1. Was it a good or bad experience?
      1. What was good or bad about it?
   2. Would you use it again?
      1. How would you feel if you had to use this tool every time you had a visit?
   3. What was it like using the iPad?
      1. Are you comfortable with the iPad technology?
   4. What aspects of the MedTrue™application did you like?
      1. Design (i.e. Look)
      2. Components (e.g. Adherence)
      3. Interface (e.g. Navigation/Usability)
   5. What aspects did you dislike?
      1. Bugs?
      2. Missing data?
      3. Hard to navigate?
2. How would you describe the MedTrue™ application to a friend or family member?
   1. On a scale of 1 to 10 (1 being low 10 being high), what is the likelihood you would recommend MedTrue to a friend or family member?
      1. What made you give that score?
3. Are there certain types of people who may have an easier or harder time using MedTrue™?
4. Why?
5. What would you change about MedTrue™?
   1. Is there anything missing that would make it more useful?
      1. Easier to use?
      2. Additional information or functionality?

Specific

1. To what extent did it help you make an accurate list of your medications?
   1. How did it help make an accurate list or not?
2. To what extent did it change your appointment or your interaction with your nurse or physician?
   1. Medication adherence?
3. Do you have any other thoughts you would like to share?

**Semi-Structured Nurse Interview Guide**

**Oral Consent**

You have been participating in a research study examining the impact of MedTrue™. MedTrue™ is a tool that is designed to facilitate gathering the best possible medication history. You recently expressed your interest in discussing your experience with MedTrue™ with a member of the study team. You can choose not to answer any questions you do not feel comfortable answering. The focus group will last about an hour and will be audio recorded.

At all times, you will know where the recorder is and if it is turned on. Once we are done with the focus group, the recording will be moved to a secure and password protected server only accessible by authorized study team members. This recording will then be deleted from the recorder. We will then transcribe the recording, removing any statements or data which could identify you. Upon checking the transcription for accuracy, we will delete the recording stored on our servers.

There are no known risks to you from taking part in this research study.

Although you may not directly benefit from this research, there is a potential benefit to people in the future as a result of information gathered in the research study.

You will not receive any compensation for participating in this research.

Please understand your participation is voluntary and you have the right to withdraw your consent or discontinue participation at any time without penalty. Specifically, your current or future employment at Geisinger will not be jeopardized if you choose not to participate.

If you have any questions about this research study you can contact Dr. Eric Wright at XXX-XXX-XXXX or Dr. Michael Gionfriddo at XXX-XXX-XXXX.

If you would like to obtain more information, offer input or discuss problems or concerns about your rights as a research participant, please call Geisinger Institutional Review Board (IRB) at XXX-XXX-XXXX or XXX-XXX-XXXX, referencing IRB # 2018-0174.

**Questions**

General

- - 1. Tell me about your experience using MedTrue™?
       1. What aspects of the MedTrue™ application did you like?
          1. Design (i.e. Look)
          2. Components (e.g. Adherence)
          3. Interface (e.g. Navigation/Usability)
       2. What aspects did you dislike?
          1. Bugs?
          2. Missing data?
          3. Hard to navigate?
    2. How would you describe the MedTrue™ application to a friend or colleague?
       1. On a scale of 1 to 10 (1 being low 10 being high), what is the likelihood you would recommend MedTrue™ to a friend or colleague?
          1. What made you give that score?
       2. Are there certain types of people who may have an easier or harder time using MedTrue™?
       3. Why?
    3. What would you change about the MedTrue™ application?
       1. Is there anything missing that would make it more useful?

1. Easier to use?
2. Additional information or functionality?

Specific

1. How did using MedTrue™ affect the process of getting a medication history and medication reconciliation?
2. Effects of workflow
3. Time
4. Workload
5. Accuracy of medication list?
6. What effect did MedTrue™ have on your conversations with patients around medication?
7. Would you want to use MedTrue™ with every patient at every appointment? If not, which patients and what frequency, and why?
8. Do you have any other thoughts you would like to share?
